# *Mycobacterium tuberculosis* Surgical Site Infection after Cardiac Surgery in the COVID-19 Era: A Case Report

**DOI:** 10.3390/idr14010013

**Published:** 2022-02-07

**Authors:** Giulia Parolari, Chiara Sepulcri, Antonio Salsano, Daniele Roberto Giacobbe, Anna Marchese, Ramona Barbieri, Antonio Guadagno, Bruno Spina, Francesco Santini, Matteo Bassetti

**Affiliations:** 1 Division of Cardiac Surgery, Ospedale Policlinico San Martino, 16132 Genoa, Italy; giulia.parolari@gmail.com (G.P.); antonio.salsano@unige.it (A.S.); francesco.santini@unige.it (F.S.); 2 DISC Department, University of Genoa, 16132 Genoa, Italy; anna.marchese@unige.it; 3 Department of Health Sciences (DISSAL), University of Genoa, 16132 Genoa, Italy; chiara.sepulcri@gmail.com (C.S.); matteo.bassetti@unige.it (M.B.); 4 Clinica Malattie Infettive, IRCCS Ospedale Policlinico San Martino, 16132 Genova, Italy; 5 Unità di Microbiologia, IRCCS Ospedale Policlinico San Martino, 16132 Genova, Italy; ramona.barbieri@hsanmartino.it; 6 Division of Pathology, IRCCS Ospedale Policlinico San Martino, 16132 Genova, Italy; antonio.guadagno@hsanmartino.it (A.G.); bruno.spina@hsanmartino.it (B.S.)

**Keywords:** *Mycobacterium tuberculosis*, sternal wound, heart surgery, surgical site infection

## Abstract

Infection of surgical wounds with acid-fast bacilli, including tubercle bacilli, is rare, and is poorly described in the literature. We present the case of a 74-year-old male who developed a sternal wound infection after cardiac surgery due to *Mycobacterium tuberculosis* complex, diagnosed post-mortem. SARS-CoV-2 infection contributed to worsened clinical conditions and surgical site infection. A high degree of suspicion to avoid unnecessary treatments and progression to severe disease with dismal prognosis is necessary in these types of infections.

## 1. Introduction

Sternal surgical wound infections after heart surgery have a cumulative incidence ranging between 0.2% and 3% overall [1], and are associated with increased morbidity, mortality, and prolonged hospitalization [2]. The most common etiological agents are coagulase-negative staphylococci and *Staphylococcus aureus* [3]. Surgical site infections caused by *Mycobacterium tuberculosis* complex after heart surgery are rare, and less than 20 cases have been reported in the literature [3]. We describe a case of surgical site infection after cardiac surgery due to *Mycobacterium tuberculosis* complex diagnosed in a patient in a tertiary care hospital in North-West Italy.

## 2. Case Report

**Figure 1:**
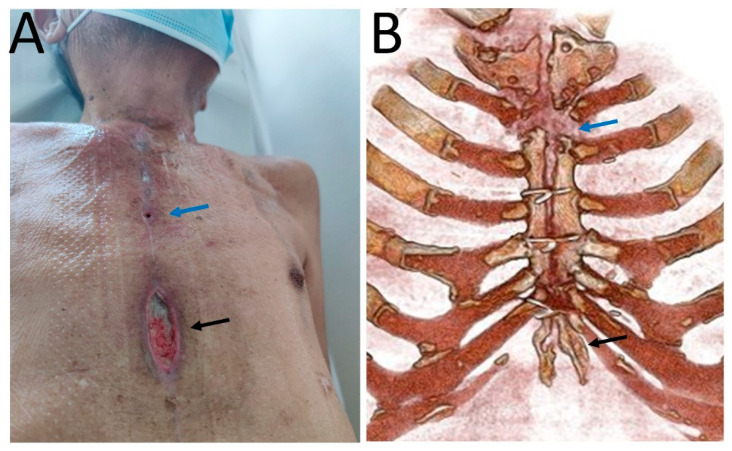
(**A**). Clinical picture of the two dehiscences in the upper and lower third of the sternotomy scar (arrows); (**B**). Three-dimensional volume-rendered CT images show sternal bone rarefaction areas in the upper and lower third of the sternum (arrows).

**Figure 2:**
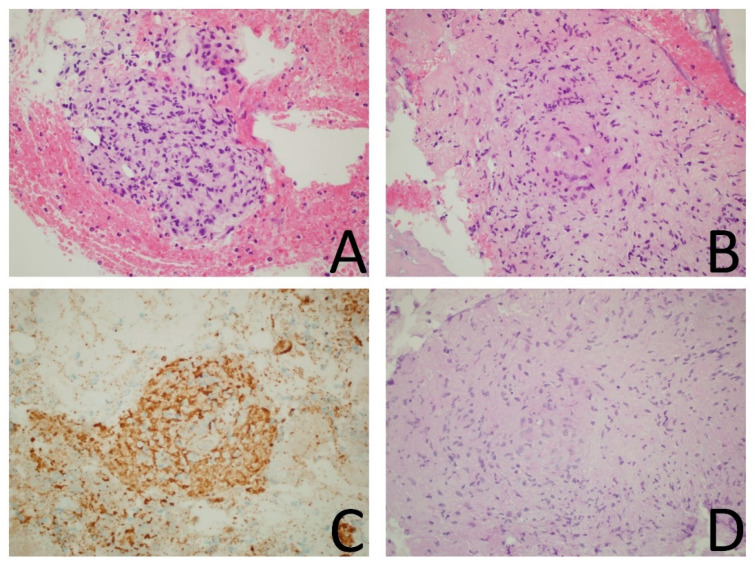
(**A**,**B**). Hematoxylin and eosin stain, original magnification 40×. The specimen was characterized by abundant fibrinoid material with numerous erythrocytes and granulocytes and by granulomatous inflammatory infiltrates with a vaguely nodular pattern composed of histiocytes and multinucleated giant cells; (**C**). CD68 stain (clone PG-M1), original magnification 40×. The histiocytes were highlighted by a CD68 stain. (**D**). Periodic acid–Schiff (PAS) stain, original magnification 40×. PAS was negative for fungal organisms. The Ziehl–Neelsen stain was also negative for *Mycobacteria* (not shown).

A 74-year-old South American male was admitted to the cardiac surgery division of a tertiary care hospital in North-West Italy in August 2020 for the onset of purulent discharge and sternal wound dehiscence 14 months after open heart surgery performed for mitral valve replacement following culture-negative mitral valve endocarditis and coronary artery bypass. Soon after the index operation, he presented superficial sternal wound dehiscence that was treated with doxycycline 100 mg twice a day with complete resolution. At that time, three consecutive wound swab cultures (APTACA Spa—Acqui Terme (AL)—Italy) were performed and produced a negative result for bacterial and fungal growth (after collection, swabs were immediately sent to the microbiology laboratory, where they were inoculated in blood, chocolate and MacConkey agar and were incubated for 48 h). The patient’s medical history included chronic sclerosing glomerulonephritis diagnosed by kidney biopsy in 2014 with kidney failure, for which he was on renal replacement therapy through peritoneal dialysis since 2017, hyperlipidaemia, hypertension, and a definitive pacemaker for atrio-ventricular block. On admission, he presented a 2 cm dehiscence on the inferior third of the median sternotomy scar without purulent exudate. C-reactive protein (CRP, assessed with Dimension Vista ^®^ 1500 Intelligent Lab—Siemens) serum levels were 67 mg/L, and white blood cell levels were 5000/mm^3^. Wound cultures were performed and produced a negative result for bacterial and fungal growth. A chest computed tomography (CT) scan was performed and showed focal bone lysis in the cranial third of the sternal body, together with pre-sternal tissue oedema. No pulmonary lesions were present. Surgical debridement was planned. In the operating room, three fistulae communicating with three subcutaneous abscesses were removed, together with the sternal scar “*en bloc*”. The wound was reconstructed with a pectoralis major mucocutaneous flap. Intraoperative specimen cultures tested positive for *Candida parapsilosis*, and therapy with voriconazole at 6 mg/kg loading doses, then 4 mg/kg twice daily, was started. Due to liver toxicity, voriconazole was interrupted 15 days after; however, the wound displayed progressive spontaneous resolution and the patient was discharged. About one month later, the patient was hospitalized for SARS-CoV-2 infection. The CT scan showed bilateral ground-glass opacities and dilated segmental and subsegmental vessels. The patient had a mild COVID-19 clinical presentation (fever, non-productive cough) with no respiratory failure (peripheral, non-invasive blood oxygen saturation measurement was 97% at room air) and for which no oxygen supplementation and no antiviral, immunomodulatory, or corticosteroid therapy was eventually necessary in line with current guidelines for mild infections [4]. Notwithstanding the mild COVID-19 presentation, he worsened clinically from the general point of view, necessitating hemodialytic treatment and progressing to cachexia. At that time, the sternal surgical wound presented two new dehiscences (Figure 1A) in the upper and lower third of the sternum, from which six swabs were performed and tested negative for bacterial and fungal growth. Vacuum-assisted closure (VAC) was applied to the wound, which showed an initial improvement. Chest CT scans showed sternal bone rarefaction areas and mediastinal lymphadenopathies (Figure 1B). With worsening general clinical conditions, the wound also worsened, with the presence of purulent and caseous discharge. A bone biopsy of the wound was performed for mycobacterial culture. Direct molecular and microscopic tests were not performed due to the paucity of the specimen. Histopathological examination revealed granulomatous inflammatory infiltrates with a vaguely nodular pattern composed of histiocytes and multinucleated giant cells (Figure 2A–E). Soon after, the patient experienced cardiac arrest and death during a hemodialysis session with hemodynamic instability not responsive to life support procedures. After the patient’s death, the bone biopsy culture tested positive for *Mycobacterium tuberculosis* complex.

## 3. Discussion

We presented a rare case of post-surgical sternal osteomyelitis following a surgical wound infection caused by *Mycobacterium tuberculosis* complex. This entity is rare among tuberculosis cases, where sternal infections represent only 1% of tubercular bone and joint infections, which in turn constitute 15% of all tuberculosis cases [1,5], The most affected areas are the sternal body and manubrium, and immunodeficiency is a leading risk factor [5]. A high level of suspicion is required for diagnosis, which is often delayed, leading to inappropriate treatments and increased morbidity and mortality.

In our case, the long and recrudescing course of the sternal wound dehiscence was pivotal to raising the suspicion on the mycobacterial origin of the infection, although the previous identification of *Candida* spp. by tissue cultures was misleading as it represented a reasonable etiological agent of chronic sternal wound infection [6], and it may have contributed to a delayed diagnosis. The clinical history was also complicated by SARS-CoV-2 infection, which possibly contributed to the general worsening of the patient’s clinical conditions and might have accelerated the course of the underlying tubercular disease. Of note, chronic renal failure per se could result in delayed wound healing [7]. Another factor possibly delaying wound healing is diabetes, which nonetheless was not present in our patient (and blood glucose and glycated hemoglobulin levels remained within normal ranges during the whole course of the disease).

Initial data on the relationship between tuberculosis and COVID-19 are currently emerging and show that global deaths due to tuberculosis rose worldwide in 2020 due to a decrease in diagnosis and access to care in the context of the interruption of essential health services due to the pandemic [8]. From the pathophysiological point of view, the association between SARS-CoV-2 and *Mycobacterium tuberculosis* complex and the associated diseases is still unclear. The add-on effect of SARS-CoV-2 infection on the immune alterations caused by tuberculosis may result in increased immunosuppression and immunological anergy, as described by Musso and colleagues [9]. Of note, this might play a role in the worsened outcome of co-infected patients. Furthermore, the immune response against SARS-CoV-2 has been shown to be hampered in vitro in patients with active and latent tuberculosis [10]. A study from Tadolini and colleagues [11] in 2020 first described a cohort of 49 active/former tuberculosis cases affected by COVID-19; of these, 28.5% suffered from COVID-19 before the diagnosis tuberculosis and two patients in total (4%) had bone involvement. The study did not infer a causal relationship between prior COVID-19 infection in tuberculosis cases, due to the fact that latent tuberculosis cases with follow up over time were not included and due to the short time between COVID-19 and tuberculosis diagnosis, with a median of 4 (range 2–10) days. A recent case report [12] suggests progression from latent to active pulmonary tuberculosis following mild COVID-19. In our case, SARS-CoV-2 infection and worsening of the sternal wound dehiscence were almost concomitant, and as such it is impossible to infer the relationship, if any, between COVID-19 and active bone tuberculosis.

## 4. Conclusions

We described a case of tubercular sternal wound infection with sternal osteomyelitis after heart surgery in a patient who also suffered from COVID-19 concomitantly. Tubercular sternal infections following cardiac surgery are rare and require a high degree of suspicion to avoid unnecessary treatments and progression to severe disease with dismal prognosis. The relationship between COVID-19 and tuberculosis is a vast and interesting topic for which large cohort studies are warranted.

## Data Availability

All available data is included in the manuscript.
